# Pony feeding management: the role of morphology and hay feeding methods on intake rate, ingestive behaviors and mouth shaping

**DOI:** 10.3389/fvets.2024.1332207

**Published:** 2024-04-12

**Authors:** Clara Bordin, Federica Raspa, Martina Greppi, Patricia Harris, Andrea Dorothea Ellis, Angela Roggero, Claudia Palestrini, Damiano Cavallini, Domenico Bergero, Emanuela Valle

**Affiliations:** ^1^Department of Veterinary Sciences, University of Turin, Grugliasco, Italy; ^2^Equine Studies Group, Waltham Petcare Science Institute, Waltham-on-the-Wolds, Melton Mowbray, United Kingdom; ^3^Mars Petcare UK, Slough, United Kingdom; ^4^Unequi Ltd., West Bridgford, United Kingdom; ^5^Department of Life Sciences and Systems Biology, University of Turin, Turin, Italy; ^6^Department of Veterinary Sciences, University of Bologna, Ozzano dell’Emilia, Italy

**Keywords:** equine, hay box, slow feeder, welfare, breed

## Abstract

In the last decade, haynets and slow feeders have been promoted as sustainable tools to improve the feeding management of horses and reduce forage waste, but little is known about their effects on ponies. Therefore, the aim of this study was to analyze the effects of different hay feeding methods on the ingestive behaviors, intake rate and mouth shaping of ponies belonging to two breed types, which are characterized by different head morphologies. Shetland type (SH, *n* = 5) and Welsh/Cob type (WC, *n* = 4) ponies were fed hay using four feeding methods: on the ground (G), a fully filled haynet (HF), a partially filled haynet (HL), and a slow-feeder hay box (HB). Head morphology was measured for each pony. Video recordings were then made to apply geometric morphometrics and to perform behavioral analysis. The intake rate was measured for each pony and each feeding method. Data obtained with geometric morphometrics were analyzed using principal component analysis (PCA) and canonical variate analysis (CVA). Behavioral data and intake rate measurements were analyzed using a mixed model, a post-hoc Tukey’s test, a Pearson’s correlation test, and a stepwise regression model. The geometric morphometrics results demonstrated that feeding method influenced mouth shaping (36% for G, 78% for HB, 77% for HF, 83% for HL, considering the total variance of shape) and affected the intake rate. Differences in mouth shaping and ingestive behaviors in SH and WC ponies also confirmed the role of morphology in feeding management. The HL proved to be the most effective tool to increase feeding consumption time when needed (5 h/kg for SH ponies and 3 h/kg for WC ponies, considering the intake time), although the HB may be the optimal choice to reduce the intake rate while maintaining a more natural posture. Future studies are suggested to fully understand how body size and morphology influence feeding in equine species.

## 1 Introduction

*Ad libitum* forage provision is not always a viable option for many reasons, especially when considering ponies and miniature horses, but most importantly it leads to a waste of resources. Forage provided on the ground is easily trampled and contaminated, resulting in a large waste of resources, both economically and in terms of sustainability. As reported by Martinson et al. (1), with loose hay provision the waste can be up to 57% of the hay provided. The percentage of waste, however, was drastically reduced with the use of a net (6%) or a slow feeder (5%). These findings were confirmed in the study by Grev et al. (2), in which several slow feeder designs were shown to be effective and paid for themselves in less than 1 year from the purchase due to the hay savings. However, it is not only important to reduce waste but also to safeguard the time spent on forage consumption to help support health, welfare, and behavior (3, 4). Stabled equines are often exposed to prolonged periods of fasting (5, 6), and as a consequence are at a greater risk of developing stereotypies and gastrointestinal diseases such as gastric ulcers (7, 8). Overweight horses and especially ponies often have to be fed a restricted intake ration in which case strategies are needed to reduce the intake rate without compromising too much of the time spent on foraging. Ponies and also miniature horses in particular are at greater risk of developing hyperlipemia and metabolic disorders (9, 10). The above has led, especially in the last decade, to the increased use of various slow feeding devices with the aim of improving the management of stabled equines from a behavioral, nutritional, and sustainable point of view. Slow feeding devices are typically reported in the literature as either a type of haynet or a ground based slow feeder. The effect of haynets with various hole sizes on feed intake and behavior has been evaluated in several studies (11–13). More recently, ground based slow feeder containers often with nets or grids to help slow down feed intake rates have been studied (2, 14). For example, a slow feeder described by Rochais et al. (14) was a bucket filled with a specific amount of hay, over which a grid was placed to mimic the effect of a haynet. However, the use of such “home-made” devices needs to be further studied as they may have effects on posture (15) and behavior that could compromise welfare. Moreover, to the best of our knowledge, the majority of the work carried out on slow feeding devices has been undertaken on horses, rather than ponies. Horses and ponies have different morphologies, and there are several differences between both horses and ponies that are often breed-related (16–19). Physical features—e.g., body weight, body size—are known to have an influence on intake rate (20, 21), and therefore further studies, particularly on ponies of different breed types, are needed in order to be able to give better and more specific management advice. This study was performed with the aim of evaluating how different methods of feeding hay (on the ground and through haynets or slow feeders) affect the intake rate and ingestive behaviors of two breed types of ponies (Shetland vs. Welsh/Cob). The geometric morphometric approach was used to characterize their head morphology to investigate how different feeding methods can alter mouth shaping, and whether there is an influence of breed type. The term “mouth shaping,” in this study refers to the shape of each pony’s mouth as it opens/closes and moves its lips to cope with the prehension of the hay administered with the different feeding methods. The hypothesis was that when the ground slow feeder was used, the ingestive behaviors and mouth shaping would be more similar to feeding directly from the ground compared to feeding from the haynets, but the intake rate would be more similar to the haynets.

## 2 Materials and methods

### 2.1 Ethics statement

This study was part of a larger research project approved by the Ethics Committee of the Department of Veterinary Sciences of the University of Turin (Prot. No. 1976; 05/07/21). Before the start of the study, the owner gave full authorization to perform the trial, with the understanding that no changes would be made to the daily routine or care of any of the ponies involved.

### 2.2 Animals

Nine healthy adult ponies used in pony game competitions were included in the study. All animals were kept in dry lot paddocks. Before the study, they were fed on the ground with long stem first cut meadow hay given three times a day. The study ponies were divided into two groups according to their morphology, passport genealogy data and their belonging to different categories during the pony games competition (the latter was based on the height of the withers, 117 cm without shoes, being used as the cutoff value, according to the Pony Club Rulebook 2022 of the Italian equestrian federation). For more details see Bordin et al. (15). Based on this, there was a Shetland breed type group (SH) which included all ponies with a withers height of less than (or equal to) 117 cm (n = 5), and a Welsh/Cob breed type group (WC) which included ponies with a withers height of more than 117 cm (*n* = 4). An example of ponies belonging to these two breed types is shown in the Supplementary Figure S1.

### 2.3 Feeding devices

The feeding devices used in this study were small perforated haynets (3.5 × 3.5 cm holes, Greedy Feeder Net, Shires Equestrian^®^, Leominster, United Kingdom) and the hay box (patent pending, Proposal No. 63358392), with the same haynets stretched over the box by a spring mechanism. The hay box was developed, designed and built at the beginning of the project by the research group (15). For research purposes two sides of the rectangular box were made of plexiglas to allow for observation of feed intake behavior. Adjustments were made for the heights of the ponies: the hay boxes were built at two heights, one with a wall height of 50 cm (for WC) and the other with a height of 45 cm (for SH). The haynets were hung so that the bottom of the haynet, in its original position, was parallel (i.e. horizontally level) to the elbow of each individual pony.

### 2.4 Study design

At the beginning of the study there was an adaptation period of 5 days (11) for all feeding devices, and for acclimatization to the equipment (e.g., 2D cameras, tripods, and operators). Subsequently, the following 2 × 4 Latin Square design was applied using the following 4 feeding methods:

1. 3 kg of hay provided on the ground (G), used as control (natural feeding position).
2. 3 kg of hay in a small perforated haynet: fully filled haynet (HF).
3. 1 kg of hay in a small perforated haynet: partially filled haynet (HL).
4. 3 kg of hay in a slow feeder hay box (HB).

The different feeding methods were presented during the normal midday meal. All other management and feeding practices were kept constant. The study was divided into two parts, the first part focused on the data collection for geometric morphometric analysis, while the second part focused on behavioral analysis.

### 2.5 Data collection

#### 2.5.1 Evaluation of the head morphometric measurements

A specific protocol was developed for the head morphometric measurements of the ponies.

**Figure 1:**
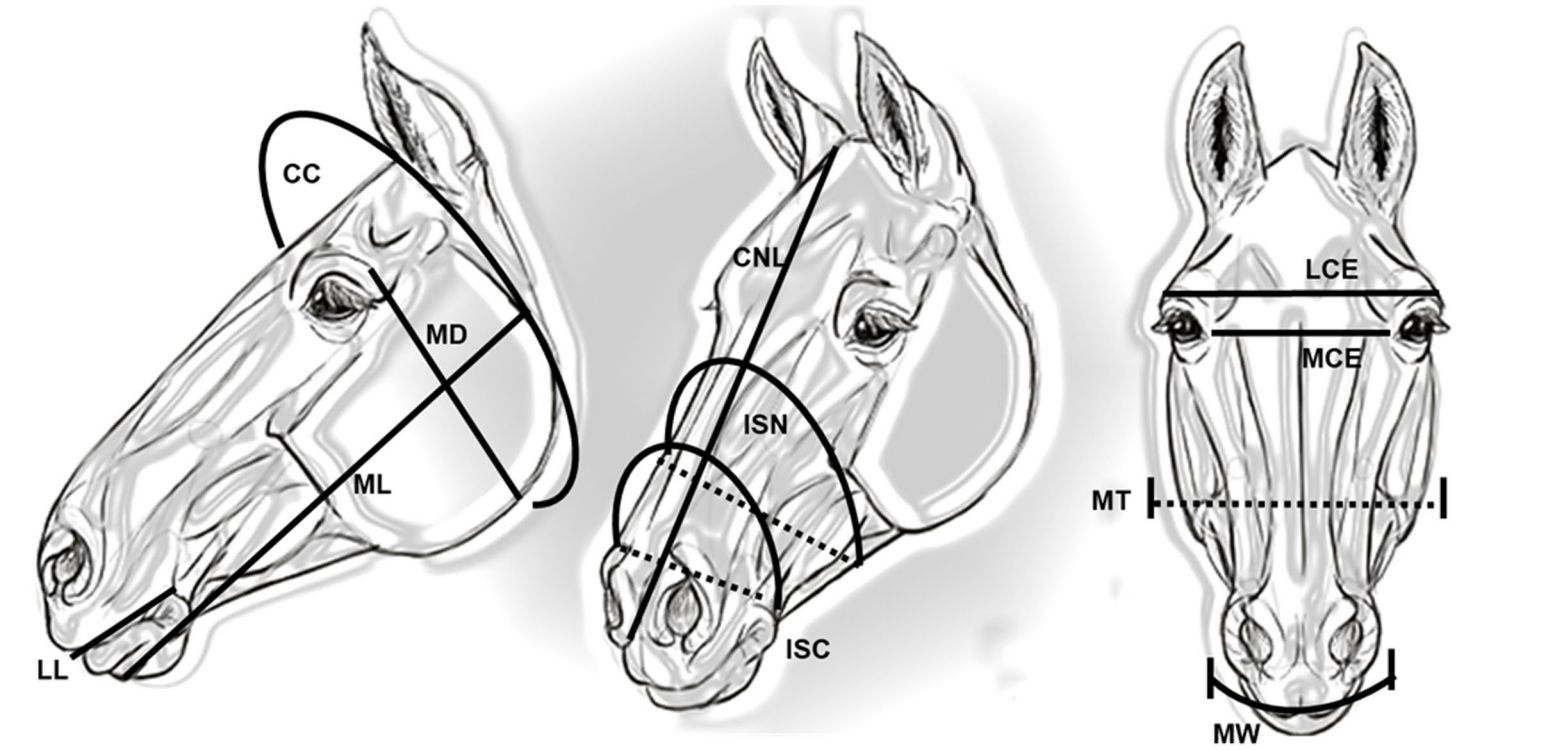
The morphometric measurements of the head. CC, cranial circumference; MD, mandibular depth; ML, mandibular length; LL, length of the lips; CNL, cranial nasal length; ISN, intermandibular space at the noseband level; ISC, intermandibular space at the chin grave level; LCE, length between the lateral corner of the eyes; MCE, length between the medial corner of the eyes; MT, mandibular thickness; MW, mouth width.

All but one of the measurements were obtained using a soft measuring tape. Mandibular width/thickness (MT) was obtained using a caliper. The cranial/nasal length (CNL) was measured from the occipital crest to the rostral point of the incisive bone and the mandibular depth (MD) was measured from the orbital notch to the widest part of the mandible (16). The width of the oral fissure and the length between the right and left commissures of the lips were measured (22) and recorded as the mouth width (MW). The following measurements (Figure 1) were added *ex novo*:

- Cranial circumference (CC), measured with the nape of the neck as the reference point, passing with the tape behind the mandibular angles and in front of the ears.
- Mandibular length (ML), measured from the chin to the mandibular angle.
- Length between the medial corners of the eyes (MCE), measured from the medial corner of one eye to the other.
- Length between the lateral corners of the eyes (LCE), measured from the lateral corner of one eye to the other.
- Length of the lips (LL), length of the lateral labial commissure.
- Intermandibular space at the level of the chin groove (ISC), measuring the distance between the mandibular branches and the nasal circumference at the same reference point.
- Intermandibular space at the noseband level (ISN), measuring the distance between the mandibular branches and the nasal circumference at the same reference point.
- Distance between mandibular angles (DMA), measured from one mandibular angle to the other.

#### 2.5.2 Video recordings

For each video recording procedure, 2D cameras (Sony HDR-CX240 with 1080p HD resolution) were used. During the video recordings performed for the geometric morphometric analysis, the cameras were placed on a tripod at a distance of 2 m from the pony and centered on its head. During the procedure of video recording for the behavioral analysis, the cameras were put on tripods that were placed parallel to the pony during the feeding period at the same (standardized) distance, height and zoom setting for each recording.

#### 2.5.3 Video analysis for mouth shaping assessment using geometric morphometrics

**Figure 2:**
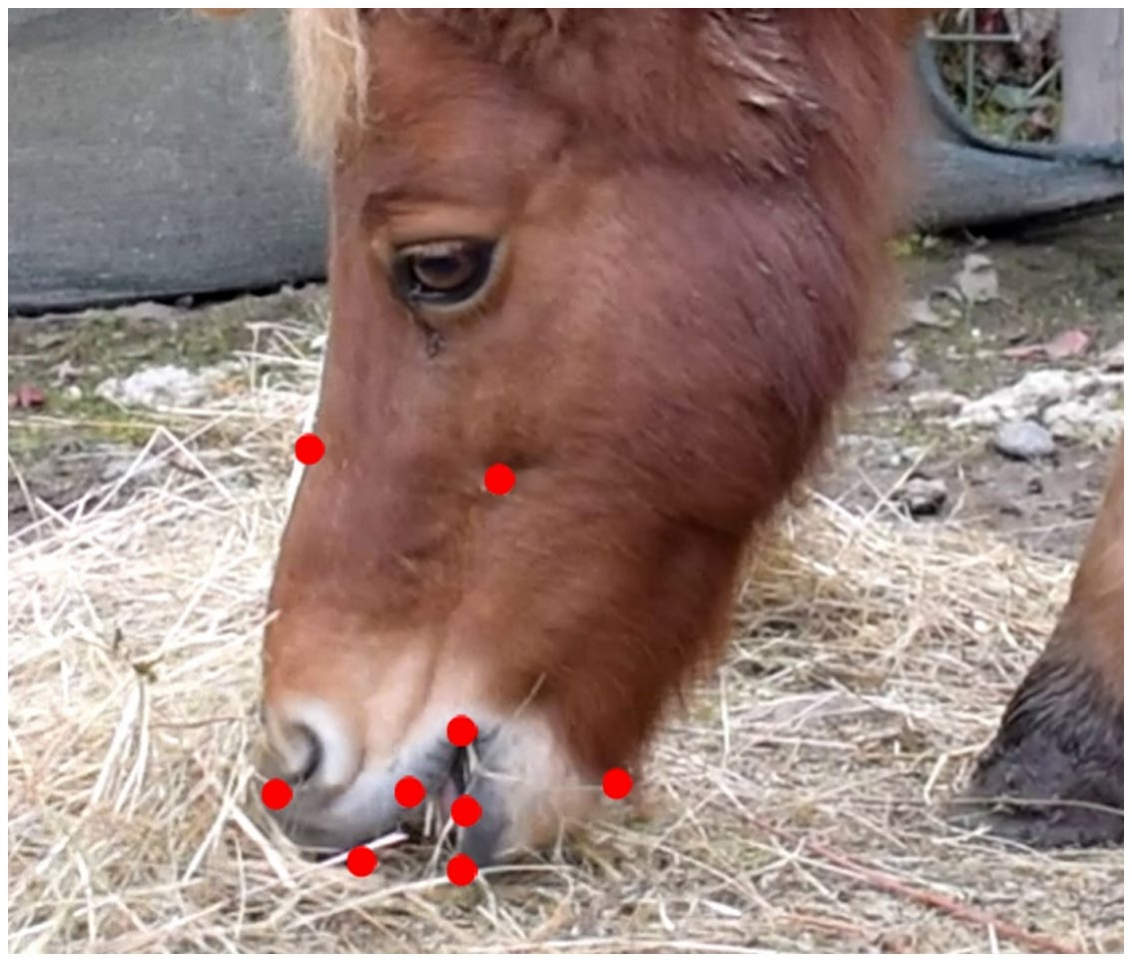
Figure of a Shetland pony with an example of the configuration used for both breed types in geometric morphometric analysis, with 9 points along the rostral part of the head using tpsDig 2.31 (24).

Each pony was video recorded in a standard posture, i.e., on its left side with its head, neck and back aligned and parallel to the camera. For each feeding method at least 30 min of video were obtained per pony during the meal. As previously described (4, 15, 23), the video recordings were analyzed by a trained operator in order to select at least 10 still frames per feeding method per pony using VLC Media Player (version 3.3.4). Subsequently, the geometric morphometric approach was applied to study the overall mouth shaping variation using a configuration of 9 points (Figure 2) placed along the rostral part of the head using tpsDig 2.31 (24). The same configuration was applied to both breed types. The points were coded as landmarks and semi-landmarks at the beginning of the trial as implemented in tpsUtil 1.82 (25). The configuration was tested for goodness of fit using tpsSmall 1.36 (26).

#### 2.5.4 Video analysis for behavioral analysis

Video recordings of each pony started 5 minutes after the meal provision via each of the feeding methods (G, HB, HF, HL). Video recordings for 15 min were made at the beginning/middle/end of the 3 h observation period, resulting in 3 videos being made per pony per feeding method. Subsequently, the 5 central minutes from each of the three video recordings were evaluated using a continuous sampling method. The videos were analyzed after an inter-observer reliability assessment by two operators trained in equine behavioral observation. A specific ethogram was developed that included the different behaviors reported in Table 1. The analysis was undertaken using BORIS, an open source software for event-logging and video coding [Behavioural Observational Research Interactive Software, version v.8.20, University of Turin, Italy (28)] (29–31). The following measurements were extrapolated from the 15 min behavioral analysis (i.e., multiplied from 15 min to 1 h): bites/h, bite and pull/h, bite and ripping/h, chews/h and biting bursts/h.

**Table 1:** Ethogram with the definition and explanation of the ingestive behaviors analyzed.

Ingestive behavior	Definition
Bite	The horse takes a bite in order to eat the hay on the ground or from the haynet or slow feeder by pulling out the forage [adapted from Ellis et al. (11)]
Bite and pull	The horse bites and moves the haynet/hay box, resulting in the haynet/container being lifted/tossed with an obvious movement along the vertical axis [adapted from Hodgson et al. (27)]
Bite and ripping	The horse bites and moves the haynet/hay box, resulting in the haynet/container being lifted/tossed (haynet/bin etc) with an obvious movement along the vertical axis [adapted from Hodgson et al. (27)]
Chewing	Number of full circular molar movements with the engagement of the masseter muscle
Biting burst	More than two consecutive prehensions before chewing

#### 2.5.5 Intake rate measurements

**Figure 3:**
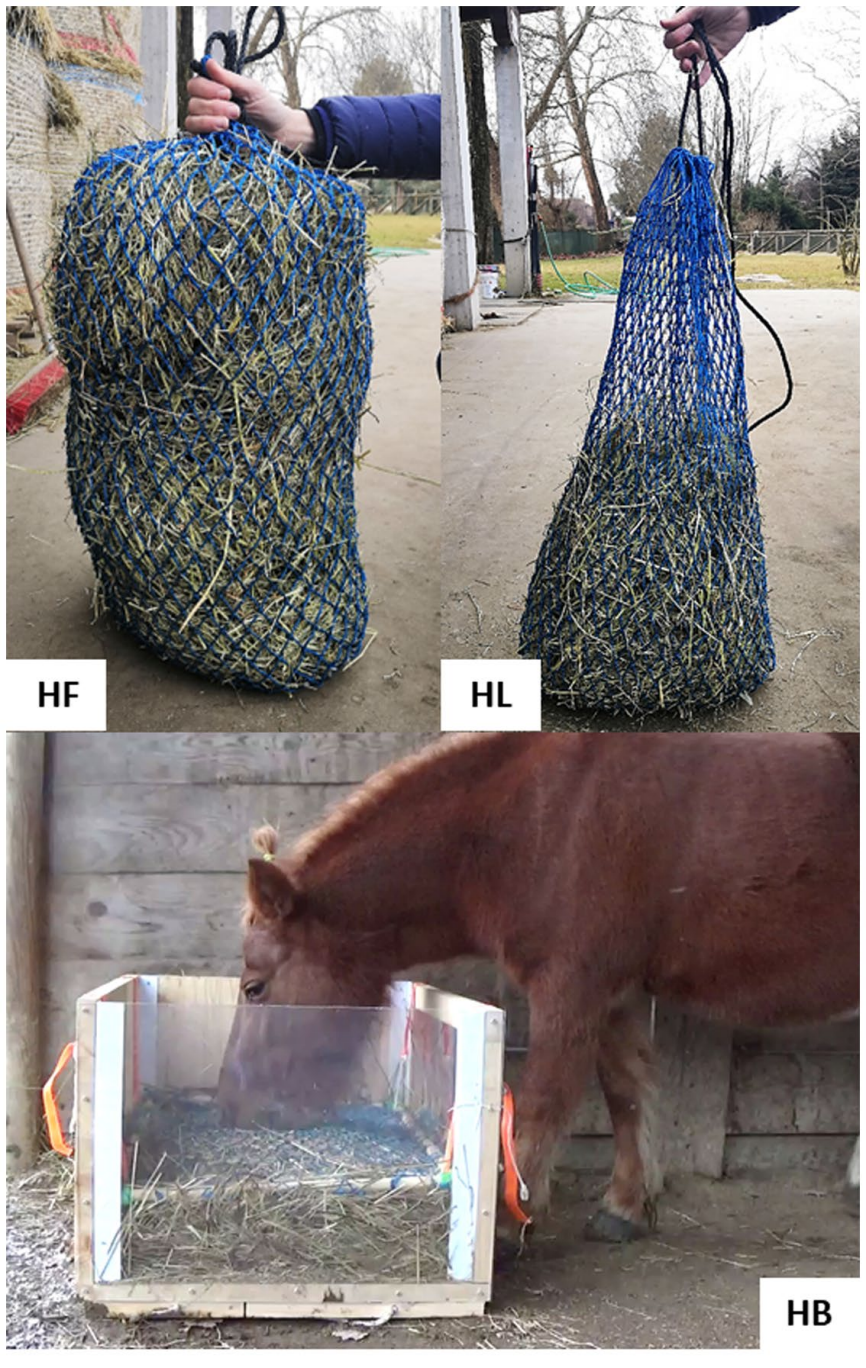
The haynet filled with 3 kg of hay, HF; the haynet filled with 1 kg of hay, HL; the hay box, HB.

The average intake rate (in g/h) for each feeding method was calculated from three repeated observations for each pony at each feeding method. This involved weighing the hay (originally 3 kg for G, HB, and HF or 1 kg for HL) (Figure 3) at the end of the 3 h observation period (or until all the hay had been consumed if earlier than 3 h) on 3 occasions. From this (making the assumption that a constant intake rate would be maintained over time or over a 24 h period) we estimated on an as fed basis (in wet matter):

- Intake rate: g/h (IN_Rate_).
- Intake time: hour/kg (IN_Time_).
- The time that would be spent (hours) on ingesting forage (hay) if fed at 1.5% BW on a dry matter basis (IN_Time_^DM1.5%BW^). The lower intake value of 1.5% BW dry matter was chosen because feeding devices are often used to increase intake time for equids on restricted diets.
- Percentage of 24 h (%) that would be spent on eating hay if given at 1.5% BW on a dry matter basis (%day^DM1.5%BW^).

From the behavioral data and intake rate measurements, the above behavioral measures were also defined per kg of hay as fed (wet matter) as:

- Bites/kg.
- Bite and pull/kg.
- Bite and ripping/kg.
- Chews/kg.
- Biting bursts/kg.

### 2.6 Data analysis

Unless stated otherwise analyses were undertaken using the SPSS Statistics v28 (IBM SPSS^©^) software and significance was set at *p* < 0.05.

#### 2.6.1 Mouth shaping data analysis

Principal component analysis (PCA) based on the selection of the first two relative warp scores, RWs1 and RWs2, was conducted using tpsRelw 1.75 (32). PCA analysis was performed to assess the mouth shaping variation in relation to the four feeding methods (G, HB, HF, and HL) and between the two breed types (SH and WC). The Canonical Variate Analysis (CVA) was used to explain the overall shape variation using all RWs (33, 34) for the following purposes:

- To assess the goodness of the classification of the two breed types considering all the ponies individually in a unique data set for each feeding method.
- To confirm the presence of morphological variation between SH and WC, evaluating the two breed types separately for each feeding method.
- To understand if there were significant differences between the ground feeding based (G and HB) and the haynet (HF and HL) methods, and consequently if the hay box could be equated to the hay provided on the ground.

#### 2.6.2 Ingestive behaviors and intake rates

Data were statistically analyzed using JMPpro v17 software (SAS Institute Inc., Cary, NC).

All data were tested for normal distribution using the Shapiro–Wilk test. Measurements were not normally distributed, and they were therefore normalized by Box–Cox transformation. Subsequently, a mixed model procedure was applied using as fixed effects the feeding methods, the breed types, and their interactions. After that, the normal distribution of the residuals was checked to confirm model adequacy. A post-hoc Tukey’s test was applied for significant interactions between individual treatments and breed types. Results are reported as medians and percentiles are given (25th and 75th). The significance was set at *p* < 0.05.

For relationships between intake rates (IN_Rate_ g/h) based on feeding methods and breed types, a Pearson’s correlation test was carried out (35), after confirming the normal distribution via the Shapiro–Wilk test. The strength of correlations (*r*-coefficient) was assessed according to Prion and Haerling (36) (if *r* = >0.90 = very strong; 0.68–0.90 = strong; 0.36–0.67 = moderate). A stepwise regression model (bidirectional type, *p*-value inclusion threshold of <0.05) was then used to evaluate whether certain head morphometric measurements could be considered as predictors of intake rate (see Figure 3).

## 3 Results

### 3.1 The effect of morphology and feeding methods on mouth shaping variation

#### 3.1.1 Mouth shaping variation

**Figure 4:**
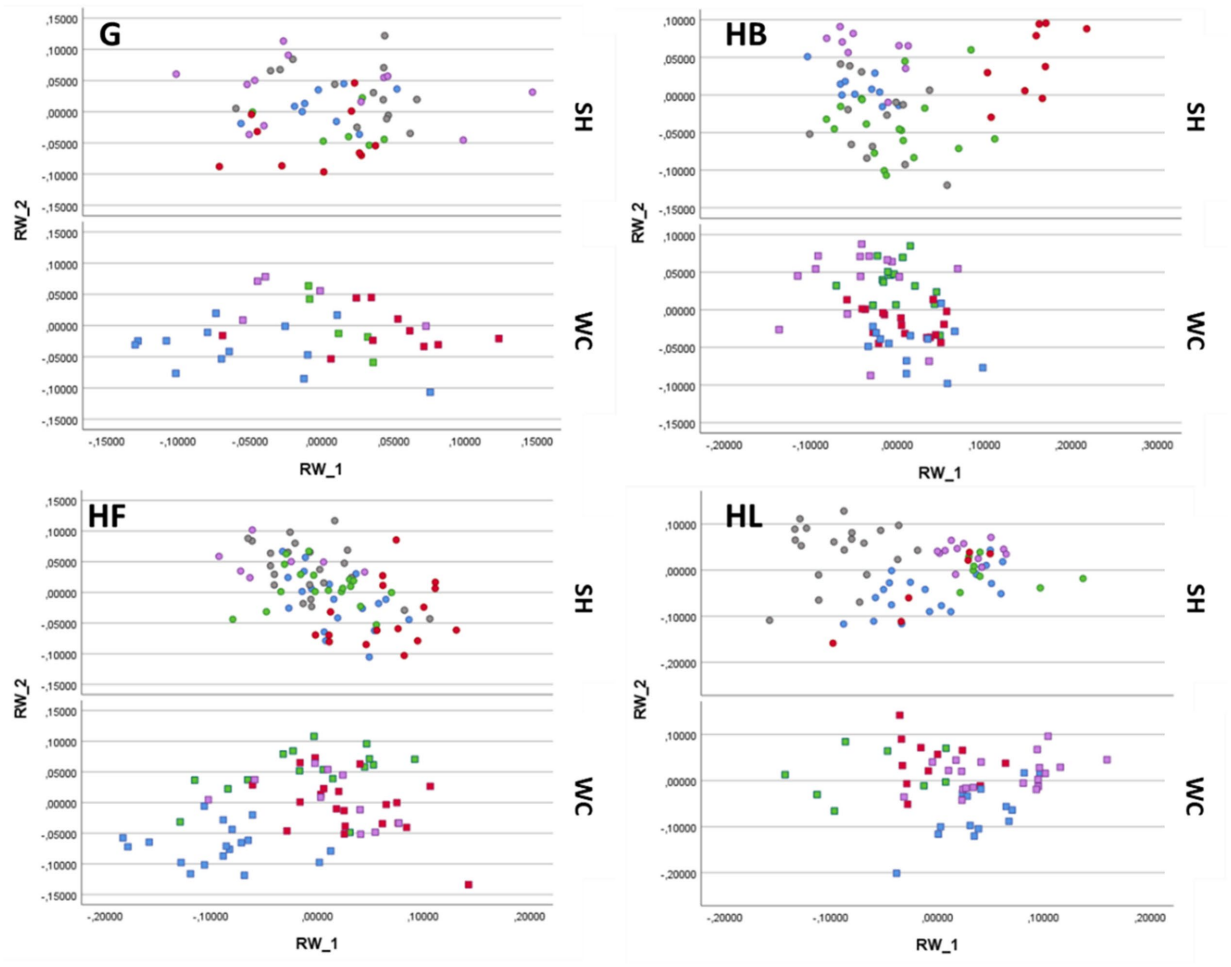
Scatterplot of the first two relative warp scores (RWs) considering the mouth shaping variation of the two breed types (SH and WC) for each feeding method: on the ground (G), fully filled haynet (HF), hay box (HB), partially filled haynet (HL). Each pony is represented by dots or squares of one color.

The PCA scatterplots (Figure 4) of the two breed types (SH and WC) for each feeding method (G, HB, HF, and HL) illustrated the variation in mouth shaping. The percentages of variance were observed to be 41.35% when the hay was provided on the ground, 45.14% with the use of the hay box, 43.51% and 44.87% when using the fully filled haynet and the partially filled haynet, respectively.

The shape variation shown by RWs 1 and 2 was relatively low, and a better representation was given when examining all the RWs together (see the CVA results in the paragraphs below). However, the results did highlight differences between the four feeding methods, as well as some similarities in the shape variation of the mouth within each breed type. Transformation grids that illustrate the morphospaces of the average value of mouth shaping for each feeding method are shown in the Supplementary Figures S2–S5.

#### 3.1.2 Classification of individuals by feeding method

**Figure 5:**
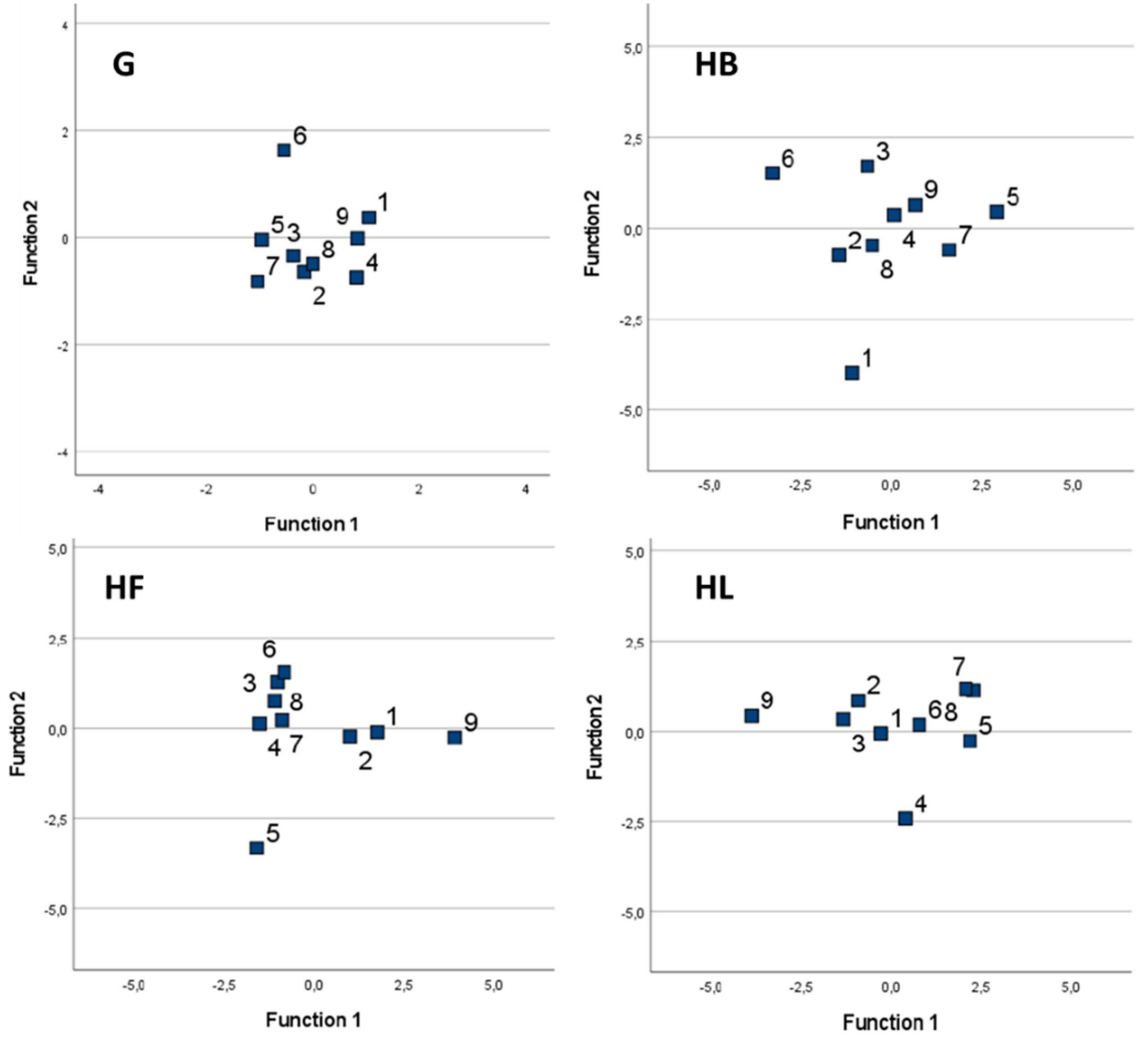
Scatterplot of the CVA for mouth shaping according to the four feeding methods: on the ground (G), fully filled haynet (HF), hay box (HB), and partially filled haynet (HL). The ponies belonging to the Shetland breed type (SH) are identified with the numbers 1, 6, 7, 8, 9, while the Welsh/Cob breed type (WC) ponies are marked with the numbers 2, 3, 4, 5.

The goodness of the classification of the ponies into the two groups for the four feeding methods was examined using all the RWs obtained from the PCA analysis of the whole dataset considering both breed types. A plot for each feeding method is shown in Figure 5, with each individual identified by a unique number. In the case of the hay provided on the ground a tighter distribution of the data was shown, with all the ponies quite close to each other, irrespective of their breed type. In this case, the percentage of groups correctly classified was low at 35.7%: reflecting little difference between all nine ponies when fed freely from the ground.

The hay box plot graphically shows a wider distribution of the individuals within the two breed types, with 78.3% of the individuals being correctly classified into their designated groups. The data from the two haynets (HF and HL) filled with different amounts of hay, also correctly classified 76.7% and 83.2% of the individuals. This is illustrated by the plots shown in Figure 5 where the fully filled haynet (HF) shows an even wider distribution compared with the more grouped data in the plot of the partially filled haynet (HL).

#### 3.1.3 Classification of breed types by feeding method

The results of the CVA analysis (Table 2) made as a variation of Hotelling’s *T*-square between the two breed types (SH vs. WC) for each feeding method (G, HB, HF, and HL) confirmed the goodness of the classification according to their morphological traits. For the hay provided on the ground 73.8% of the original group was correctly assigned to the two breed types, while for the slow feeding devices—HB, HF, and HL—the percentages were 80.8%, 80.1%, and 81.5%, respectively. Although the percentage was lower for ground feeding, it was still high at nearly 75% correct classification suggesting that there were differences between the two groups of ponies with all four feeding methods, reflecting differences in the mouth morphology of each breed type.

**Table 2:** The CVA results showing the group membership accuracy considering the four feeding methods (G, HB, HF, and HL)^1^ for each breed type (SH and WC)^2^.

Feeding method	Group	Predicted group membership		% Total
		SH	WC	
G	SH	68.6	31.4	100
	WC	27.3	72.7	100
HB	SH	75.0	25.0	100
	WC	16.7	83.3	100
HF	SH	74.4	25.6	100
	WC	17.2	82.8	100
HL	SH	83.3	16.7	100
	WC	32.1	67.9	100

^1^G, ground; HB, hay box; HF, fully filled haynet; HL, partially filled haynet. ^2^SH, Shetland; WC, Welsh Cob.

#### 3.1.4 Mouth shaping comparing ground feeding methods (G and HB) to haynets (HF and HL)

CVA analysis showed that there was a clear distinction in terms of mouth shaping between ground feeding (G and HB) and haynet feeding (HF and HL), such that 78.7% of the individuals’ mouth shaping could be correctly classified into the two groups when comparing their data from the combined ground and haynets feeding methods as shown in Table 3.

**Table 3:** The CVA results showing the accuracy of group membership considering the subdivision into ground feeding methods and haynets (G + HB, HF + HL)^1^.

Group	Predicted group membership		% Total
	G + HB	HF + HL	
G + HB	80.9	19.1	100
HF + HL	24.5	75.5	100

^1^G, ground; HB, hay box; HF, fully filled haynet; HL, partially filled haynet.

### 3.2 Influence of different feeding methods and morphologies on intake rate and ingestive behaviors

#### 3.2.1 Ingestive behaviours and intake rate according to feeding methods and breed

The various feeding methods significantly influenced all the feeding behaviors measured in this study (*p* < 0.0001, Table 4). There was a significant difference (*p* < 0.05) between the two breed types (SH and WC ponies) for many of the behaviors. However, there were no significant differences between breed groups for bites/h, bites and pulls/kg, biting burst/kg and chews/h. An interaction between the fixed effects (*p* < 0.001), was found suggesting a relationship between the feeding methods and breed type on pony intake rate. Table 5 reports the median intake rate and the other indices estimated from it with the results of the mixed model procedure: IN_Time_, IN_Time_^DM1.5%BW^, %day^DM1.5%BW^. Again, as found with the feeding behavior analysis, the feeding method had a significant effect on the IN_Rate_ (*p* < 0.001). The IN_Rate_ for G was the highest for both breed types (IR = 532 g/h and 865 g/h, for the SH and WC respectively) whereas the HL gave the lowest IN_Rate_ (190 g/h for SH ponies and 333 g/h for WC ponies). The IN_Rate_ for HB or HF was also lower than that for G. The results for measures derived from intake rates (Table 4) showed a similar pattern. Breed type and therefore the morphology, was shown to have a significant influence (*p* < 0.05) only on IN_Rate_ and IN_Time_ (h/kg).

**Table 4:** Ingestive behaviors expressed by ponies according to the four feeding methods (G, HB, HF, and HL)^1^ and the two breed types (SH and WC)^2^.

Behavior	Breed	Feeding methods, median (25th–75th percentiles)				*p*-value^**^		
		G, 3 kg of hay	HB, 3 kg of hay	HF, 3 kg of hay	HL, 1 kg of hay	Feeding methods	Breed	Feeding methods × breed
Bites/h	SH	160 (149–176)^C^	1,012 (756–1,174)^B^	1,406 (1,180–1,674)^A^	836 (764–836)^B^	<0.001^*^	0.67	0.13
	WC	192 (116–192)^C^	886 (808–1,237)^B^	1,692 (964–1,736)^A^	906 (760–1,436)^B^			
Bites/kg	SH	321 (236–380)^C^	4,066 (2,760–5,225)^B^	4,724 (3,611–6,552)^A^	4,092 (3,253–6,967)^AB^	<0.001^*^	0.02^*^	0.69
	WC	206 (132–256)^C^	2,208 (1,454–256)^B^	3,371 (2,619–3,687)^A^	3,164 (2,704–4,120)^AB^			
Bites and pull/h	SH	n/a	60 (43–173)^B^	456 (271–491)^A^	400 (244–436)^A^	<0.001^*^	0.02^*^	<0.001^*^
	WC	n/a	306 (257–382)^B^	360 (328–828)^A^	462 (388–516)^A^			
Bites and pull/kg	SH	n/a	308 (156–522)^B^	1,464 (1,128–1,681)^A^	1,723 (1,290–2,295)^A^	<0.001^*^	0.32	<0.001^*^
	WC	n/a	718 (541–918)^B^	1,205 (681–1,930)^A^	1,617 (1,171–1,808)^A^			
Bites and ripping/h	SH	n/a	8 (3–89)^A^	0 (0–0)^B^	8 (0–52)^B^	<0.001^*^	0.03^*^	<0.001^*^
	WC	n/a	98 (50–233)^A^	24 (12–140)^B^	68 (64–116)^B^			
Bites and ripping/kg	SH	n/a	26 (15–225)^A^	0 (0–0)^C^	47 (0–168)^B^	<0.001^*^	0.03^*^	<0.001^*^
	WC	n/a	242 (110–449)^A^	58 (23–254)^C^	209 (148–328)^B^			
Biting burst/h	SH	n/a	156 (112–169)^B^	216 (189–224)^A^	204 (172–228)^A^	<0.001^*^	0.03^*^	<0.001^*^
	WC	n/a	234 (181–251)^B^	188 (172–310)^A^	218 (188–268)^A^			
Biting burst/kg	SH	n/a	557 (448–706)^B^	742 (715–843)^B^	978 (670–1,387)^A^	<0.001^*^	0.12	0.01^*^
	WC	n/a	469 (410–642)^B^	583 (357–767)^B^	687 (618–849)^A^			
Chews/h	SH	3,612 (3,368–3,888)^A^	1,128 (1,091–1,313)^C^	1,242 (1,015–2,160)^B^	1,104 (700–1,412)^C^	<0.001^*^	0.44	0.45
	WC	3,676 (3,596–3,896)^A^	1,152 (1,070–1,585)^C^	1,464 (1,442–1,934)^B^	1,088 (1,084–1,492)^C^			
Chews/kg	SH	7,418 (5,314–8,530)^A^	4,879 (3,475–5,775)^B^	4,645 (3,637–7,713)^B^	6,220 (4,296–9,200)^AB^	<0.001^*^	0.05^*^	0.79
	WC	4,504 (3,876–5,656)^A^	3,072 (1,852–4,260)^B^	3,536 (2,949–5,445)^B^	3,540 (3,127–5,046)^AB^			

Data expressed as medians (25th–75th percentiles). h, hour; kg, kilograms; n/a, not applicable. ^1^G, ground; HB, hay box; HF, fully filled haynet; HL, partially filled haynet. ^2^SH, Shetland; WC, Welsh Cob. ^**^Mixed model procedure with feeding method, breed type, and their interaction as fixed effects. ^*^Statistical significance: *p* < 0.05.

**Table 5:** Intake rates (g hay/h) and related measures according to the four feeding methods (G, HB, HF, HL)^1^ and the two breed types (SH and WC)^2^.

Behavior	Breed	Feeding methods, median (25th–75th percentiles)				*p*-value		
		G, 3 kg of hay	HB, 3 kg of hay	HF, 3 kg of hay	HL, 1 kg of hay	Feeding methods	Breed	Feeding methods × breed
IN_Rate_ (g/h)	SH	532 (417–741)^A^	293 (197–330)^B^	286 (244–335)^B^	190 (150–257)^C^	<0.001^*^	0.01^*^	0.65
	WC	865 (663–997)^A^	468 (336–586)^B^	455 (356–505)^B^	333 (276–411)^C^			
IN_Time_^DM1.5%BW^ (h)	SH	5.3 (3.6–7.4)^C^	11.7 (7.9–13.8)^B^	10.1 (8.9–12.2)^B^	14.7 (11.1–19.7)^A^	<0.001^*^	0.12	0.67
	WC	4.4 (3.8–6.2)^C^	8.8 (5.9–12.1)^B^	8.5 (6.8–11.6)^B^	11.7 (10.1–15.3)^A^			
% day^DM1.5%BW^ (%)	SH	21.8 (14.8–31.0)^C^	48.8 (33.0–57.7)^B^	42.2 (37.2–50.7)^B^	61.1 (46.2–81.9)^A^	<0.001^*^	0.12	0.68
	WC	18.4 (15.6–25.8)^C^	36.6 (24.6–50.2)^B^	35.6 (28.4–48.5)^B^	48.7 (41.9–63.4)^A^			
IN_Time_ (h/kg)	SH	1.9 (1.3–2.4)^C^	3.4 (3–5)^B^	3.5 (3–4.1)^B^	5.3 (3.9–6.7)^A^	<0.001^*^	0.01^*^	0.69
	WC	1.2 (1–1.5)^C^	2.2 (1.7–3)^B^	2.2 (1.9–2.8)^B^	3.0 (2.4–3.6)^A^			

Data expressed as medians (25th–75th percentiles). h, hour; g, grams of hay; kg, kilograms of hay; %, percentage of 24 h. ^1^G, ground; HB, hay box; HF, fully filled haynet; HL, partially filled haynet. ^2^SH, Shetland; WC, Welsh Cob. ^*^Statistical significance: *p* < 0.05.

#### 3.2.2 Head morphometric measurements correlated to intake rate (g hay/h)

The results reported in Table 6 show that the greater the mouth width, the greater the intake rate was for SH ponies feeding especially from HB (*p* < 0.05, *r* = 0.71, strong), but also from HF (*p* < 0.05, *r* = 0.59, moderate). However, the head morphometric measurement that showed the strongest correlation with intake rate was the intermandibular space length at the noseband level. The length of the intermandibular space at the noseband level was negatively correlated for SH ponies fed with HB (*p* < 0.05, *r* = −0.72, strong) and HF (*p* < 0.05, *r* = −0.51, moderate), and for WC ponies fed with HL (*p* < 0.05, *r* = −0.70, strong). On the contrary, the intermandibular space circumference at the noseband level, had a positive correlation with the intake rate for SH when HF was used (*p* < 0.05, *r* = 0.67, moderate). The results obtained for the intermandibular space measured at the chin groove level, showed that for SH ponies the space length was negatively correlated with the intake rate when they were fed with both the HB (*p* < 0.05, *r* = −0.65, moderate) and HF (*p* < 0.05, *r* = −0.54, moderate), whereas its circumference was negatively correlated with the intake rate when fed on the ground (*p* < 0.05, *r* = −0.55, moderate). WC ponies demonstrated a positive correlation with intake rate when looking at the results for the length between the medial corners of the eye when fed on the ground (*p* < 0.05, *r* = 0.52, moderate), and also for the length between the lateral corners of the eye when fed with HL (*p* < 0.05, *r* = 0.62, moderate).

**Table 6:** Relationship between head morphometric measurements and intake rate (g hay/h) according to breed type (SH and WC)^1^ and feeding method (G, HB, HF, and HL)^2^ (Pearson’s correlation coefficient *r*: grey shading = strong; bold = moderate).

Head morphometric measurements (cm)	Intake rate (g hay/h)															
	G, 3 kg of hay				HB, 3 kg of hay				HF, 3 kg of hay				HL, 1 kg of hay			
	SH		WC		SH		WC		SH		WC		SH		WC	
	*r*	*p*	*r*	*p*	*r*	*p*	*r*	*p*	*r*	*p*	*r*	*p*	*r*	*p*	*r*	*p*
CNL	0.34	0.20	0.31	0.22	0.52	0.12	0.13	0.76	0.40	0.12	0.26	0.39	0.40	0.15	0.39	0.22
CC	0.39	0.14	0.40	0.12	0.50	0.14	−0.10	0.81	0.37	0.16	0.15	0.63	0.39	0.15	0.28	0.38
MD	−0.09	0.75	−0.13	0.62	0.36	0.31	−0.31	0.46	0.19	0.49	−0.11	0.71	0.20	0.48	−0.31	0.33
ML	**−0.55**	**0.03** ^*^	0.26	0.32	−0.27	0.45	0.47	0.24	−0.30	0.25	0.40	0.19	−0.30	0.27	**0.63**	**0.03** ^*^
MT front	0.35	0.19	0.13	0.63	−0.25	0.49	0.36	0.38	−0.21	0.43	0.14	0.64	−0.21	0.46	0.35	0.26
MT middle	0.32	0.22	0.44	0.08	−0.14	0.70	0.29	0.49	−0.28	0.29	0.28	0.35	−0.10	0.72	**0.61**	**0.03** ^*^
MCE	**−0.47**	**0.07** ^*^	**0.52**	**0.03** ^*^	0.20	0.59	−0.04	0.92	−0.29	0.27	0.13	0.68	0.12	0.67	0.42	0.18
LCE	−0.28	0.29	0.44	0.08	0.31	0.38	0.30	0.47	0.24	0.38	0.29	0.34	0.09	0.75	**0.62**	**0.03** ^*^
MW	−0.38	0.15	−0.24	0.35	0.71	0.02^*^	0.38	0.35	**0.59**	**0.02** ^*^	0.24	0.44	0.46	0.09	0.18	0.58
LL	**−0.50**	**0.05** ^*^	0.32	0.21	0.45	0.19	−0.18	0.67	0.21	0.44	0.10	0.75	0.21	0.45	0.16	0.62
ISC length	−0.15	0.58	0.34	0.18	**−0.65**	**0.04** ^*^	−0.53	0.19	**−0.54**	**0.03** ^*^	−0.11	0.71	−0.48	0.07	−0.15	0.64
ISC circumference	**−0.55**	**0.03** ^*^	0.43	0.09	0.54	0.11	−0.13	0.77	0.41	0.12	0.14	0.66	0.27	0.34	0.28	0.37
ISN length	0.06	0.83	−0.26	0.32	−0.72	0.02^*^	−0.54	0.17	**−0.51**	**0.05** ^*^	−0.41	0.16	−0.50	0.06	−0.70	0.01^*^
ISN circumference	−0.30	0.26	0.09	0.74	0.52	0.12	−0.13	0.76	**0.67**	**0.00** ^*^	0.07	0.81	0.28	0.31	0.02	0.95
DMA	0.25	0.35	0.42	0.10	0.02	0.95	0.11	0.80	0.03	0.91	0.12	0.71	−0.04	0.90	0.40	0.19

cm, centimeters; *p*, *p*-value; CNL, cranial nasal length; CN, cranial circumference; MD, mandibular depth; ML, mandibular length; MT, mandibular thickness; MCE, length between the medial corner of the eyes; LCE, length between the lateral corner of the eyes; MW, mouth width; LL, length of the lips; ISC, intermandibular space at the chin grave level; ISN, intermandibular space at the noseband level; DMA, distance between mandibular angles. ^1^SH, Shetland; WC, Welsh Cob. ^2^G, ground; HB, hay box; HF, fully filled haynet; HL, partially filled haynet. ^*^Statistical significance: *p* < 0.05.

Moreover, the mandibular length was negatively correlated with the intake rate (*p* < 0.05, *r* = −0.551, moderate) when SH ponies were fed with the hay on the ground, i.e., the shorter the mandible of the pony the lower the intake rate g/h. In WC ponies, on the other hand, there was a positive correlation between this measurement and the IR when fed with HL (*p* < 0.05, *r* = 0.63, moderate). Furthermore, the WC ponies fed via the HL showed a positive correlation between the mid-mandibular thickness and the IR (*p* < 0.05, *r* = 0.61, moderate).

The measurement of the length of the lips showed a negative correlation with the intake rate in SH ponies fed on the ground (*p* < 0.05, *r* = −0.50, moderate).

#### 3.2.3 The potential of head morphology to predict intake rates (g hay/h) when providing loose hay on the ground (G)

Table 7 shows the results of the stepwise regression model evaluating which of the head morphometric measurements could be considered as a predictor(s) for estimating the Intake rate in g/h (INRate) of ponies when fed on the ground. This showed that the cranial circumference (*p* = 0.001), the intermandibular space circumference at the chin groove level (*p* = 0.041) and the mouth width (*p* = 0.047) were the best predictors of intake rate.

**Table 7:** Head morphometric measurements showing significant predictability for estimating intake rate (IN_Rate_ g/h) in 9 ponies fed hay on the ground.

Parameter (cm)	Significance value
Cranial circumference	0.001^*^
Intermandibular space circumference at the chin groove level	0.041^*^
Mouth width	0.047^*^

cm, centimeters. ^*^Statistical significance: *p* < 0.05.

Subsequently, using these results the following prediction expression was obtained:


$$\mathrm{Intake}\;\mathrm{rate}\;\left(g\;\mathrm{hay}/h\right)\;\mathrm{of}\;\mathrm{hay}\;\mathrm{when}\;\mathrm{fed}\;\mathrm{on}\;\mathrm{the}\;\mathrm{ground}$$

$$13.85766661+\left[26.609660973\times \mathrm{cranial}\;\mathrm{circumference}\;\left(\mathrm{cm}\right)\right]$$

$$+\left[\begin{array}{l} -21.51201485\times \mathrm{intermandibular}\;\mathrm{space}\;\mathrm{circumference}\;\\ \mathrm{at}\;\mathrm{the}\;\mathrm{chin}\;\mathrm{groove}\;\mathrm{level}\;\left(\mathrm{cm}\right) \end{array}\right]$$

$$+\left[-52.82805392\times \mathrm{mouth}\;\mathrm{width}\;\left(\mathrm{cm}\right)\right]$$

This prediction expression was based on the intake rate (IN_Rate_ g/h) of all ponies (SH and WC) as estimated over 3 h.

## 4 Discussion

This is the first published study to measure the effect on mouth shaping, ingestive behaviors and intake rate when using slow feeding devices in ponies of two breed types (SH vs. WC). Previous studies using a geometric morphometric approach have evaluated the influence of slow feeding management devices on the body posture of horses (37) and the back and neck posture of ponies (15). Using geometric morphometry of the neck and back in our previous study (15), supported the classification of these ponies into two breed types (SH vs. WC). The current study provided further support for this through the CVA findings reported in Table 2, which showed a clear difference between the breeds. Geometric morphometrics however, is not only applicable to the study of body posture, in fact, this technique has also been used to characterize head morphology enabling comparisons among and within breeds, as well as between horses and donkeys (16, 38–40). Applying this technique to facial changes (i.e., mouth shaping) during ingestion, this study demonstrated that all four feeding methods resulted in different mouth shaping in the two breeds avaluated (Figure 4), and they also influenced how ponies prehended the hay, and consequently intake rates. Furthermore, when considering the total variance of the shape obtained with the CVA analysis taking all nine ponies together (Figure 5) it was found that both SH and WC ponies used their mouths similarly when fed on the ground, highlighting little differentiation (36%). On the contrary, the use of partially filled haynets led to the highest variance of mouth shaping between individuals (83%), followed by the hay box (78%) and the fully filled haynet (77%). This shows that all the ponies reacted very individually when restrictive feeding management methods were applied. When focusing on the analysis of mouth shaping by the feeding devices, the results (Table 3) showed that although HB resulted in differences in mouth shaping between the animals, these were still similar to feeding from the ground and therefore could be effectively pooled with G, and similar mouth shaping was also seen for both haynet treatments. This was also shown in our previous study of neck posture (15). However, if horse owners do not adjust the height of the haynet according to the height of the pony, as was done in this study, there may well be different effects on mouth shaping.

The use of both types of slow feeding devices (i.e., hay boxes and haynets) significantly altered the ingestive behavior of the ponies (Table 4). Feeding from the HF led to the greatest increase in Bite/h and Bite/kg compared to *ad libitum* ground feeding for both breed types (Table 4). Previous studies have shown a similar effect of increased intake rate and thereby increased feeding time (12, 13). This is not surprising given that as a consequence of the haynet being filled the surface becomes more or less convex and “tight,” thus affecting the ponies’ ability to bite and grab the hay and requiring them to modify their mouth shaping in order to pull out the forage from the net. Hodgson et al. (27) also reported changes in bite rates and pulling pressures exerted by horses between different forage sources (hay or haylage) which would also have been affected by haynet surface and fill level. The increase in Bites/kg was in line with previous studies and in this study, we also observed a reduction in Chews/kg (G to HB, −34% and −32%; G to HF, −37% and −21%; G to HL, −16% and −21%; for SH and WC ponies respectively) in the feeding devices. Ellis et al. (11), also reported numerous small bites from small perforated haynets before chewing. As the ponies in this study were smaller, even more bites occurred and each bite may have already released smaller pieces of feed than would have been pulled out by larger horses. SH ponies had a higher number of bites/kg with all restricted feeding methods and especially a higher number of chews/kg when compared to WC ponies, showing a strong effect of head morphology. Indeed, it has been reported that small animals have a higher chewing frequency due to a shorter chewing cycle (41). As expected, the haynets (HF and HL) showed the highest values of bite and pull/h and bite and pull/kg. This behavior was exacerbated by their attachment (i.e., haynets are hung) which made them very mobile. It is possible that these behaviors could lead to postural and dental problems over time due to the pressure exerted (27). When considering the hay box slow feeder (HB), our findings showed that its use resulted in the highest values of “ripping” behavior (bite and ripping/h and bite and ripping/kg). These are the only ingestive behaviors for which we obtained a significant effect of the feeding device, the breed, and also an interaction between them. It is worth underlining that the WC ponies, in particular, showed a high expression of these behaviors when feeding from both the haynets and hay box. The hay used was relatively brittle so ripping from haynets was perhaps less necessary for ponies, whereas a ripping action was required to pull hay out from the flat surface of the hay box. Only two other studies reported feed intake behavior on flat restricted surfaces (42, 43). Hongo and Akimoto (43) showed that a considerable amount of pressure had to be exerted by horses to “pluck” grass from a flat artificial grass feeder. Moreover, the much higher incidence of Bite and ripping in WC compared to SH may have been a breed temperament effect (44), with WC ponies showing frustration due to restricted access by lifting up the feeding restrictor in the hay box. Other studies have reported the development of frustration behaviors when evaluating the use of haynets, especially double layer haynets (12, 27).

In estimating daily time budgets from these results, it is obvious that the HL provided only 1 kg of hay while the HB, HF, and ground all provided 3 kg. Comparing only the feeding methods that provided 3 kg of hay, the study showed that while the HF reduced the intake rate the most for both SH and WC compared to feeding *ad libitum* from the ground, the HB values were not as different suggesting that it was similarly useful in extending consumption time by reducing the intake rate. Among the three feeding methods which provide 3 kg of hay, HB was the method that increased the %day^DM1.5%BW^ for hay, i.e. the time that would be spent in foraging activities (when feeding forage in DM at 1.5% BW over a 24h period). This was true for both SH and WC ponies (with increases of 49 and 37%day^DM1.5%BW^, respectively over ground feeding) almost doubling the values obtained with G (22 and 18%day^DM1.5%BW^, respectively). However, the best results in terms of increase in feeding consumption time were gained with HL (61 and 49%day^DM1.5%BW^ for SH and WC ponies, respectively) with the caveat that the “up-calculation” from 1 kg may have a slightly higher inaccuracy. These findings highlight how much this type of feeding management can facilitate a more natural time budget, in terms of the percentage of time spent feeding during the day.

Direct comparisons between studies on feed intake behavior can be difficult due to the fact that, in addition to the equipment used to feed, forage type and its fracture properties affect prehension and chewing times (12). Furthermore, all published comparative studies have used large horses rather than ponies. However, a basic conversion of our results to mean times to ingest 1 kg of fresh matter (FM) can be compared to previous studies (Table 8). The comparisons reported in Table 8 show a really important difference when assessing the intake rates of small and medium sized ponies, with much slower intake rates (min/kg FM), especially from feeding devices due to their smaller mouth anatomy. When converting the data from other authors using average reported body weight to IN_Time_^DM1.5%BW^, as would be appropriate for horses on a weight loss regime, the feed intake times move much closer together again (Table 8). Therefore, on average the smaller breeds, although ingesting much less per hour, still spend approximately the same time on ingestive behavior if fed around 2% of BW in DM. This natural behavioral urge to spend a mean of 12 ± 2 h on foraging behavior (3, 45), as observed in feral and stabled horses, has been maintained in the larger breeds, although their intake rates are much faster due to their size. Therefore, more research on intake rates in ponies needs to be undertaken in the future. Moreover, the results obtained would suggest that when trying to manage individuals prone to weight gain or predisposed to metabolic disorders (3, 9, 46), HL may be the optimal solution to reduce intake rate and increase feeding consumption time. However, the HL would have to be refilled more frequently than the HB to prevent prolonged periods without forage consumption, which may not be practically possible for the majority of horse owners/carers. In addition, based on recent discoveries on the effects of haynets on posture and behaviors, the use of a ground slow feeder, such as the one developed and tested in this study, may be preferable as it not only increases the foraging time budget but also results in a more natural feeding posture. Despite these promising findings, further studies are needed to find the best solution for the use of a ground slow feeder when managing different sized ponies and horses together.

**Table 8:** Comparison of mean feed intake times for forages fed on the ground or with various feeding devices.

Authors	Mean BW^*^ (kg)	Feeder	Device opening size (cm)	Haynet/feeder fill (kg)	Forage	IN_Time_ (min/kg FM)	IN_Time_^DM1.5%BW^ (h)
Ellis et al. (11)	619	Eliminet^™^	1.8 × 1.8	6	Hay/Haylage	33	6.4
	619	Furlongs^™^ Anti-Vice Net	3 × 3	6	Hay/Haylage	29	5.6
	619	Shires Haylage Net	3 × 3	6	Hay/Haylage	28	5.4
	619	Large Hole Net	9 × 9	6	Hay/Haylage	25	4.8
	619	Shires Haylage Net	3 × 3	6	Hay	33	6.4
	619	Shires Haylage Net	3 × 3	6	Haylage	26	5.0
Glunk et al. (13)	513	Small Hole Net,	3.20	5	Hay	67	9.5
	513	Medium Hole Net	4.40	5	Hay	55	7.8
	513	Large Hole Net	15.20	5	Hay	46	6.6
	513	Loose from Ground		5	Hay	40	5.7
Ellis et al. (12)	585	Shires Haylage Net	3 × 3	6	Hay	39	6.3
	585	Shires Haylage Net	3 × 3	3	Hay	30	4.9
	585	Double Shires Haylage Net	3 × 3	3	Hay	68	11.1
	585	Triple Shires Haylage Net	3 × 3	3	Hay	78	12.7
Hodgson et al. (27)	520	Shires Haylage Net	3 × 3	3	Hay	45	6.5
	520	Shires Haylage Net	3 × 3	3	Haylage	26	5.2
	520	Shires Haylage Double Net	3 × 3	4	Hay	40	5.8
	520	Shires Haylage Double Net	3 × 3	5	Haylage	24	4.8
Hallam et al. (42)	627	Haynet	9 × 9	6	Mixture hay	21	3.7
	627	Ground Feedbox with haynet/bars across	6 × 6	6	Hay (80%) & straw	39	6.8
This Study	158	Loose from Ground (SH)		6	Hay brittle	77	5.2
	158	Shires Haylage Net (SH)	3 × 3	6	Hay brittle	137	9.4
	158	Shires Haylage Net (SH)	3 × 3	3	Hay brittle	213	13.6
	158	Hay Box (SH)	3 × 3	6	Hay brittle	141	10.2
	220	Loose from Ground (WC)		6	Hay brittle	118	4.7
	220	Shires Haylage Net (WC)	3 × 3	6	Hay brittle	215	8.3
	220	Shires Haylage Net (WC)	3 × 3	3	Hay brittle	310	13.0
	220	Hay Box (WC)	3 × 3	6	Hay brittle	232	8.6

BW, body weight; cm, centimeters; kg, kilograms of hay; min, minutes; h, hours; FM, fresh matter; n/a, not applicable; SH, Shetland; WC, Welsh Cob. ^*^Reported medians and BW are estimated according to horse type.

In our study head morphology was evaluated with the aim of finding possible correlations with intake rate and discovering possible predictors of the intake rate of ponies when fed on the ground. Interestingly, mouth features in terms of width (i.e., mouth width) were positively correlated with the intake rate of SH ponies, specifically when fed from HB (*p* < 0.05, *r* = 0.71) and HF (*p* < 0.05, *r* = 0.59), thus the two slow feeding devices filled with 3 kg of hay. Therefore, for very small ponies it may be necessary to adjust haynet sizes to muzzle width after further research. The intake rate of the ponies decreased significantly with increases in their intermandibular space length at the noseband level. This occurred for WC ponies fed with HL (*p* < 0.05, *r* = −0.70) and SH ponies fed with HB (*p* < 0.05, *r* = −0.72). These results, although preliminary as they were obtained from a small number of individuals, suggest that head morphology should be taken into consideration when designing restricted feeding devices for horses and ponies. Some traits of the head will influence intake behavior more than others and this needs further evaluation, including in a wide range of horses and ponies. Finally, with respect to the head morphometrics measured in this study, we have tentatively proposed an equation that includes the cranial circumference, the intermandibular space circumference at the chin groove level and the mouth width to predict the intake rate in ponies fed on the ground (paragraph 3.2.3). This may be useful in predicting an individual animal’s potential intake rate when provided with *ad libitum* forage, in order to better manage its feeding and consequently, according to its needs, choose the best way to provide hay (e.g., haynets, slow feeders). Individual palatability, appetite and temperament will also play a role. The equation obtained is therefore a starting point, for future research.

## 5 Conclusion

This research project had some limitations due to the small number of individuals involved. Despite this, the two slow feeding devices (haynets and the hay box) were shown to influence various aspects including the expression of ingestive behaviors and consumption time. Furthermore, the study highlighted that the hay box, could be an optimal solution for restrictive forage feeding management, by allowing the owners and caretakers to increase the time budget for feeding while maintaining a more natural posture, and reducing forage waste. However, each animal is unique, and for those that need the most reduction in intake rate, partially filled haynets that are replaced as required to prevent animals from spending more than 5 h (especially during the day) without forage may be the best option. Finally, this study highlights the important role that morphology can play with respect to equine ingestive behavior and nutrition. Therefore, we recommend that further studies be carried out on the feeding management of individuals as characterized by different body sizes and morphologies.

## Data availability statement

The raw data supporting the conclusions of this article will be made available by the authors, without undue reservation.

## Ethics statement

The animal study was approved by Ethical Committee of the Department of Veterinary Sciences of the University of Turin (Prot. No. 1976; 05/07/21). The study was conducted in accordance with the local legislation and institutional requirements.

## Author contributions

CB: Investigation, Writing – original draft. FR: Conceptualization, Methodology, Writing – review & editing. MG: Investigation, Writing – original draft. PH: Conceptualization, Funding acquisition, Methodology, Writing – review & editing. AE: Conceptualization, Methodology, Writing – review & editing. AR: Conceptualization, Data curation, Formal analysis, Methodology, Visualization, Writing – review & editing. CP: Conceptualization, Data curation, Formal analysis, Methodology, Visualization, Writing – review & editing. DC: Conceptualization, Data curation, Formal analysis, Methodology, Visualization, Writing – review & editing. DB: Project administration, Supervision, Writing – review & editing. EV: Conceptualization, Methodology, Writing – review & editing.
