# Supplementary material for: Pony feeding management: the role of morphology and hay feeding methods on intake rate, ingestive behaviors and mouth shaping
Source: Front Vet Sci. 2024 Apr 12;11:1332207. doi: 10.3389/fvets.2024.1332207 (PMC11046934; doi:10.3389/fvets.2024.1332207)
Supplement: Supplementary file 1 [file Data_Sheet_1.DOCX]

Supplementary Material

Clara Bordin^1^, Federica Raspa^1^*, Martina Greppi^1^, Patricia Harris^2^, Andrea Dorothea Ellis^3^, Angela Roggero^4^, Claudia Palestrini^4^, Damiano Cavallini^5^, Domenico Bergero^1^, Emanuela Valle^1^

*** Correspondence:** Federica Raspa: [federica.raspa@unito.it](mailto:federica.raspa@unito.it)

# Supplementary Figures and Tables

##
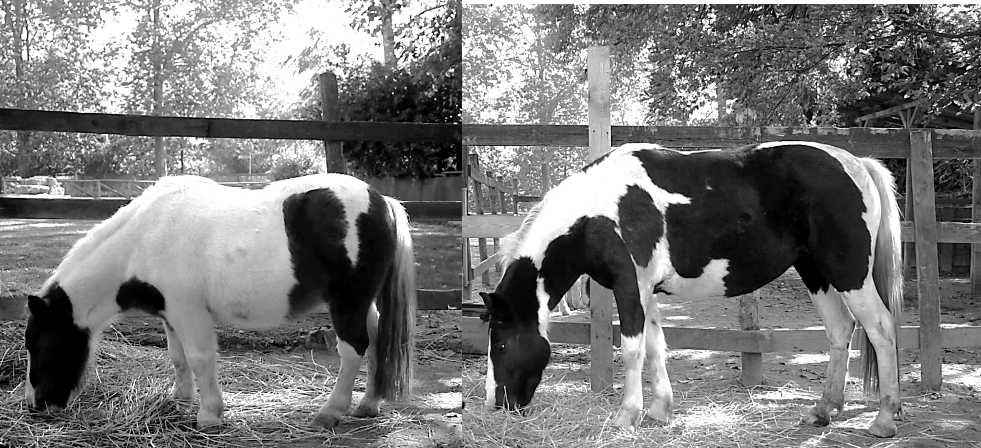
Supplementary Figures

**Supplementary Figure 1.** Figure of a Shetland breed type pony (left) and a Welsh/Cob breed type pony (right).


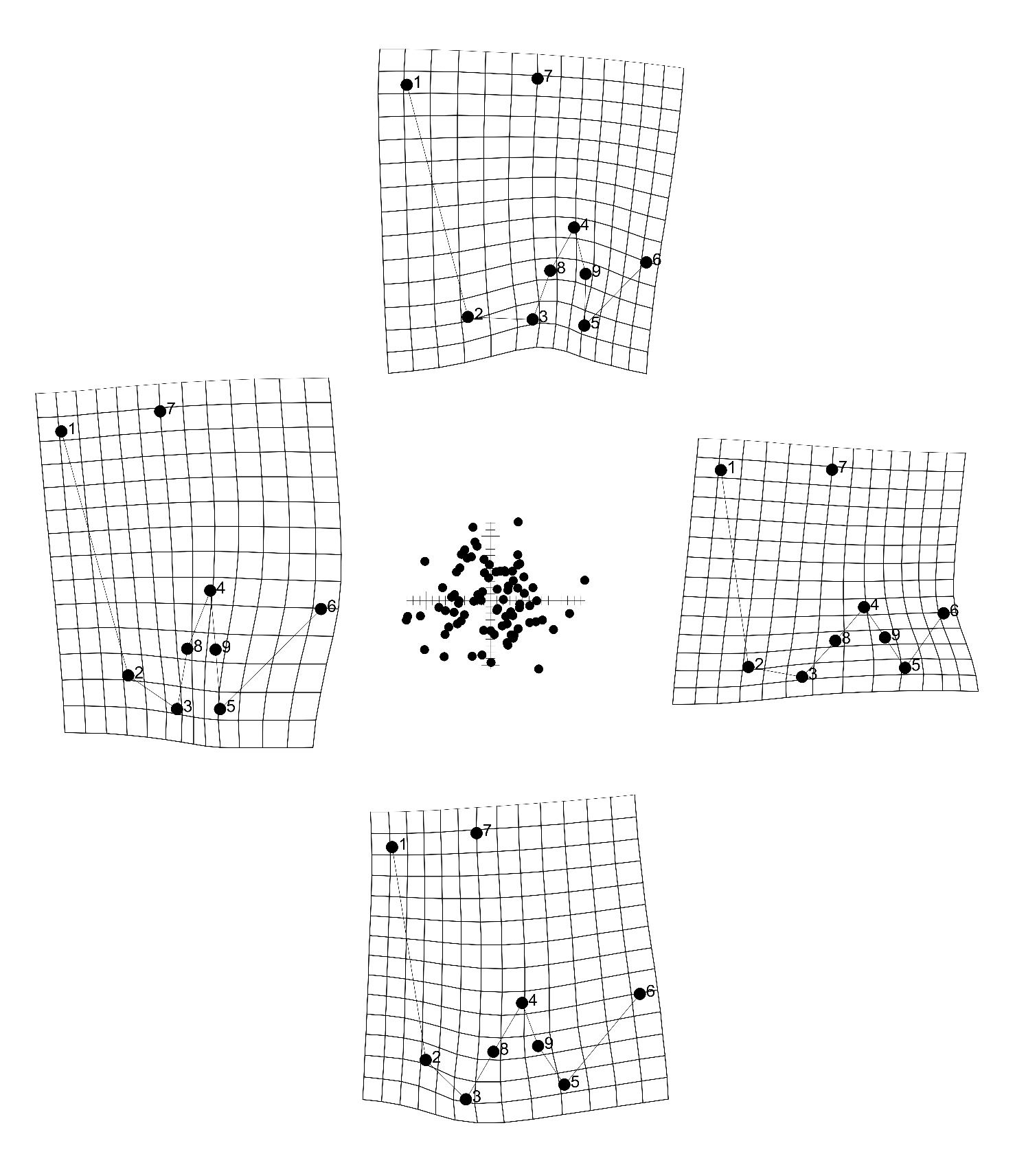
**Supplementary Figure 2.** Transformation grids representing the average value of "mouth shaping" for the hay on the ground (G). The transformation grids refer to RW_1 (X) and RW_2 (Y), their placement on the cartesian plane is casual.


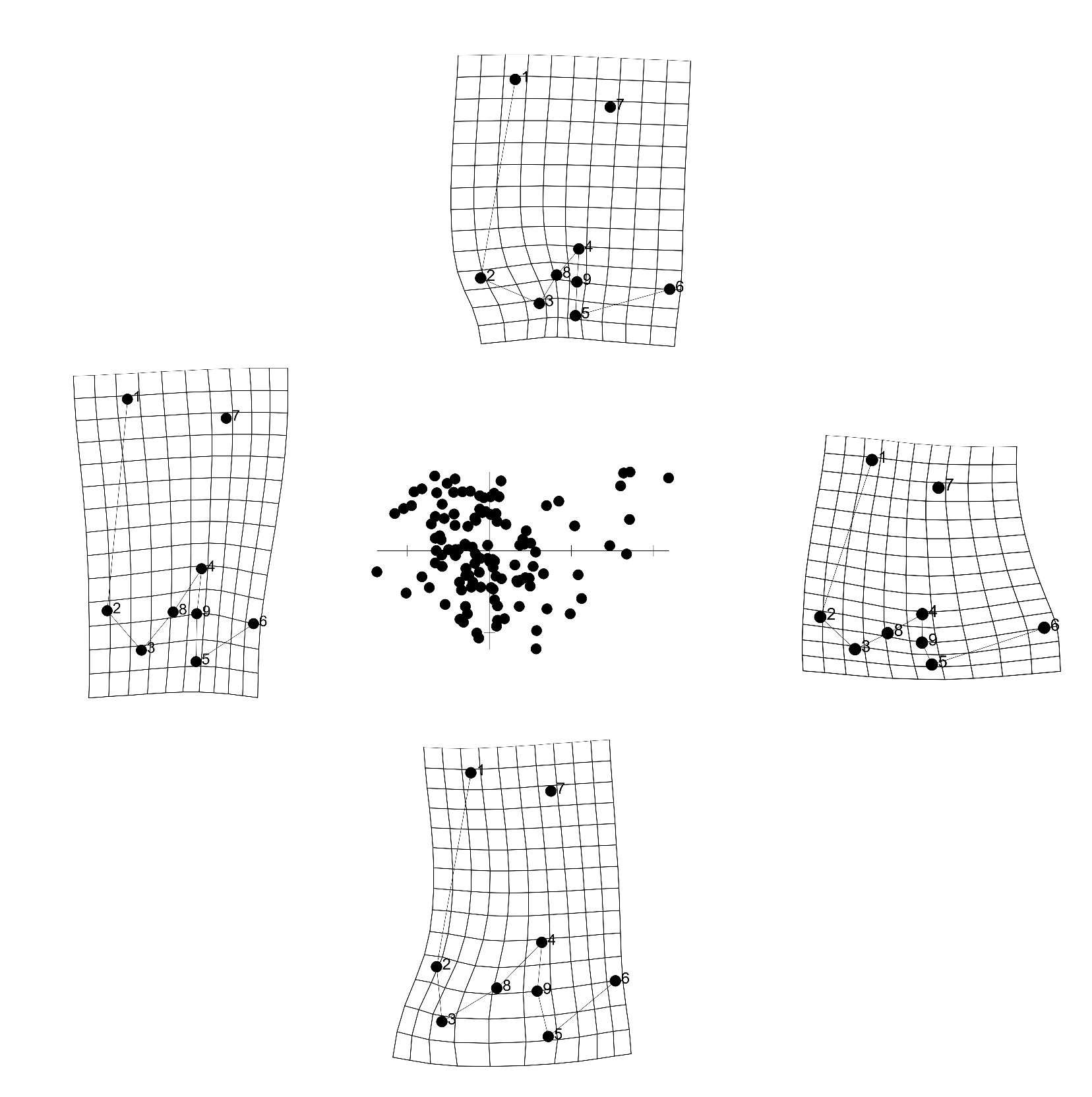
**Supplementary Figure 3.** Transformation grids representing the average value of "mouth shaping" for the hay box (HB). The transformation grids refer to RW_1 (X) and RW_2 (Y), their placement on the cartesian plane is casual.


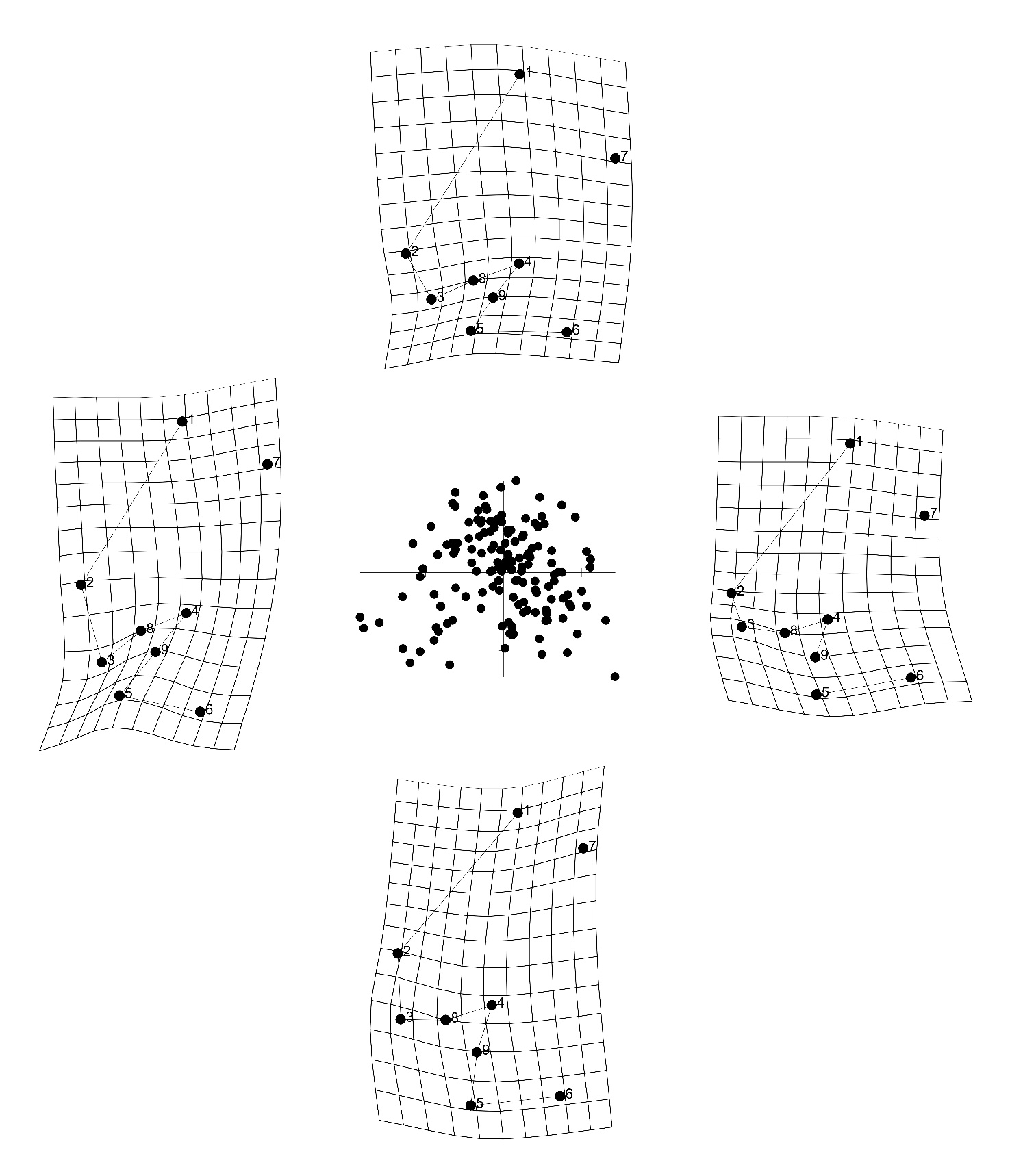
**Supplementary Figure 4.** Transformation grids representing the average value of "mouth shaping" for the fully filled hay net (HF). The transformation grids refer to RW_1 (X) and RW_2 (Y), their placement on the cartesian plane is casual.

**Supplementary Figure 5.**
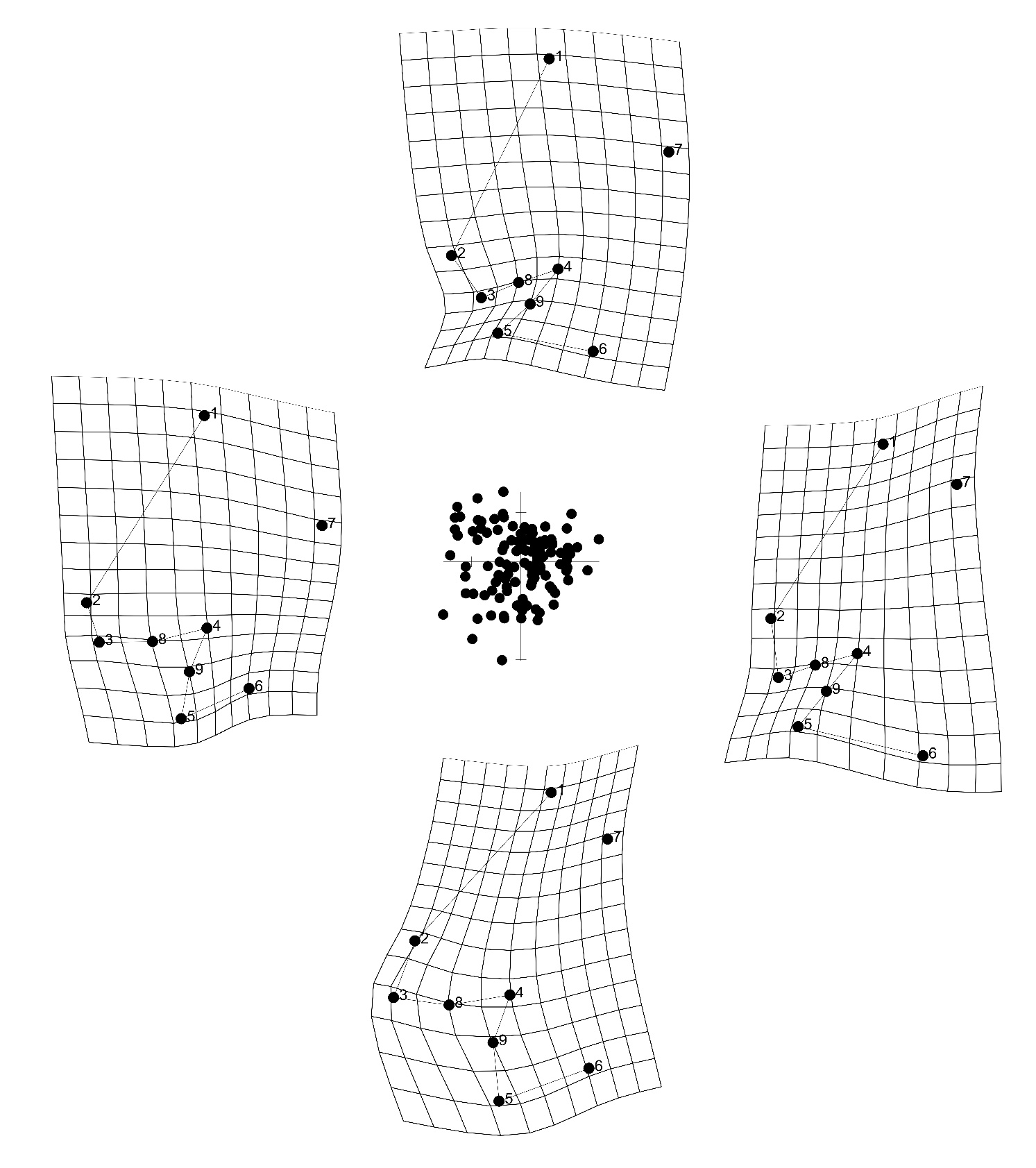
Transformation grids representing the average value of "mouth shaping" for the partially filled hay net (HL). The transformation grids refer to RW_1 (X) and RW_2 (Y), their placement on the cartesian plane is casual.


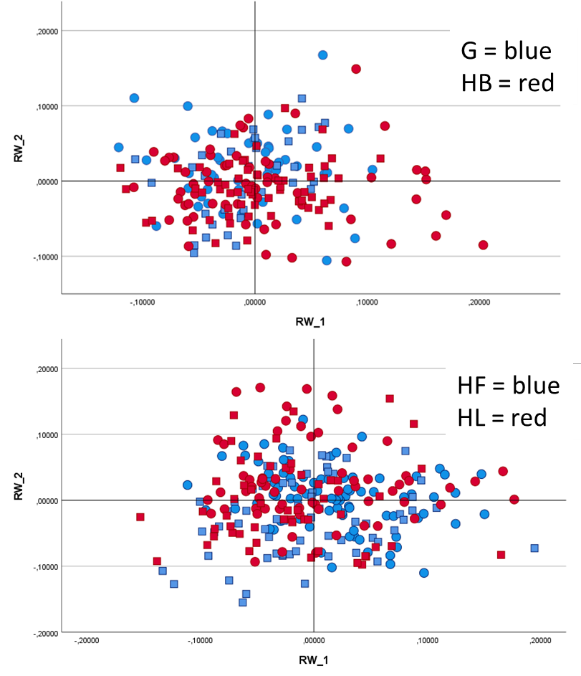


**Supplementary Figure 6.** PCA plot which represents the variation between the two subdivided groups (G + HB) and (HF+ HL) for the shape of the mouth according to the breed type. The ponies belonging to the Shetland breed type (SH) are identified with the dots, while the Welsh/Cob breed type (WC) ponies are identified with squares.
